# Diet and lifestyle behaviours simultaneously act on frailty: it is time to move the threshold of frailty prevention and control forward

**DOI:** 10.1186/s12889-024-18639-y

**Published:** 2024-04-20

**Authors:** Shan-lan Yang, Lei Wu, He-lang Huang, Lang-lang Zhang, Yi-xin Chen, Sheng Zhou, Xiu-xiu Chen, Jiao-feng Wang, Chao-bao Zhang, Zhi-jun Bao

**Affiliations:** 1 Department of Gerontology, Shanghai Key Laboratory of Clinical Geriatric Medicine, Research Center on Aging and Medicine, Huadong Hospital Affiliated to Fudan University, Fudan University, 200040 Shanghai, P. R. China; 2 Shanghai Institute of Infectious Disease and Biosecurity, Fudan University, 200040 Shanghai, P. R. China; 3 Jiangxi Provincial Key Laboratory of Preventive Medicine, School of Public Health, Nanchang University, 330006 Nanchang, P. R. China

**Keywords:** Frail, Diet, Life behavior, Decision tree, Prevalence study

## Abstract

**Background:**

To analyse the association among the simultaneous effects of dietary intake, daily life behavioural factors, and frailty outcomes in older Chinese women, we predicted the probability of maintaining physical robustness under a combination of different variables.

**Methods:**

The Fried frailty criterion was used to determine the three groups of “frailty”, “pre-frailty”, and “robust”, and a national epidemiological survey was performed. The three-classification decision tree model was fitted, and the comprehensive performance of the model was evaluated to predict the probability of occurrence of different outcomes.

**Results:**

Among the 1,044 participants, 15.9% were frailty and 50.29% were pre-frailty; the overall prevalence first increased and then decreased with age, reaching a peak at 70–74 years of age. Through univariate analysis, filtering, and embedded screening, eight significant variables were identified: staple food, spices, exercise (frequency, intensity, and time), work frequency, self-feeling, and family emotions. In the three-classification decision tree, the values of each evaluation index of Model 3 were relatively average; the accuracy, recall, specificity, precision, and F1 score range were between 75% and 84%, and the AUC was also greater than 0.800, indicating excellent performance and the best interpretability of the results. Model 3 takes exercise time as the root node and contains 6 variables and 10 types, suggesting the impact of the comprehensive effect of these variables on robust and non-robust populations (the predicted probability range is 6.67–93.33%).

**Conclusion:**

The combined effect of these factors (no exercise or less than 0.5 h of exercise per day, occasional exercise, exercise at low intensity, feeling more tired at work, and eating too many staple foods (> 450 g per day) are more detrimental to maintaining robustness.

**Supplementary Information:**

The online version contains supplementary material available at 10.1186/s12889-024-18639-y.

## Introduction

Frailty is a gradual age-related non-specific decline of the physiological system, which is primarily characterised by increased vulnerability, reduced anti-stress ability, and decreased ability to maintain balance, resulting in a series of adverse events, such as hospitalisation, falls, disability, and death, which seriously reduces the quality of life of older individuals and increases the burden of family care and the pressure on social, medical, and health resources [1–3]. Population aging and longevity are objective trends in human social development. With the continuous acceleration of the global aging process, the change in population structure increases the proportion of the frail population, and the frailty of the older individuals is becoming increasingly obvious.

Frailty is an unavoidable phenomenon in older adults and an intermediate state between health and death [4]. According to one survey, the prevalence of frailty in the older population in China is 12.8%, and the prevalence of frailty in hospitals and nursing institutions is even higher at 22.6% and 44.3%, respectively, which is consistent with the results of foreign studies [5–7]. Frailty in the older adults is sex-specific, with a higher prevalence in female than in male in both pre-frailty and frailty [8], which is related to changes in the endocrine system; in particular, the sharp decline in hormone levels in female after menopause plays an important role in sarcopenia [9].

There is a lot of research on frailty influences and interventions, especially at the level of dietary nutrition and exercise; for example, anti-inflammatory diets can counteract the effects of unfavourable factors on frail [10], and resistance training performs well in frail interventions [11]. However, there are still some issues that need to be solved: first, the current exploration of frailty in the Chinese older population has been explored at the provincial, municipal, and district levels, but has not formed a national vision; second, previous studies have mostly explored the relationship between a single-dimensional factor and frailty, but have not explored at multiple levels; third, most of the existing studies focus on the intervention after the diagnosis of frailty, assuming that if the diet and living behaviours can be standardised before the occurrence of frailty, the incidence of frailty can be reduced, the treatment of frailty can be moved forward to prevention, and the economic burden of patients and the medical burden of hospitals can be reduced. Hence, based on the above realities and limitations, this study focused on the simultaneous effects of dietary intake and lifestyle behaviours on frailty in older Chinese female population.

## Materials and methods

### Study participants and sample size

The study participants were female residents of five provinces, Fujian, Jiangxi, Shaanxi, Henan, and Hebei, which are divided into northern and southern China by the Qinling-Huaihe River line. Multistage stratified cluster sampling was carried out according to the proportion of the resident female population, gross domestic product level, and age distribution ratio in the five provinces.

### Inclusion criteria

Women (≥ 60-year-old) who actively participated in this research activity and provided all information necessary for the research.

### Exclusion criteria

Severe diseases, such as tumours, autoimmune diseases, vital organ failure, disability, psychiatric abnormality, noncompliance, and inability to participate in the study.

### Sample size

Based on the prevalence of frailty in community-dwelling older adults in China (12.8%), it is proposed that the number is the positive probability π, the test level (*α*) = 0.05 (bilateral), the allowable error (*δ*) = 0.2π, and on the basis of the calculated sample increased by 20%, according to the formula $n=\left(\frac{{Z}_{\alpha /2}}{\delta }\right)\times \pi \times \left(1-\pi \right)$, the final sample size is approximately 785 people, with a minimum sample number of each province being 157 people. The study was approved by the Ethics Committee of the Second Affiliated Hospital of Nanchang University and informed consent was obtained from all participants.

## Research method

### Information collection

We designed a questionnaire for the participants, which mainly included the following three points: basic information (education level, marital status, current or pre-retirement occupation), dietary intake (staple food, vegetarian dishes, meat dishes, fruits, seasonings, and water intake), and life behaviours (dressing, sleep, exercise, and family emotion).

### Frailty judgment

The frailty of study participants was assessed based on the Fried frailty phenotype, which describes frailty as a decline in body function associated with aging, by assessing weight, exercise, fatigue, walking, and grip strength, and meeting three or more of the criteria to be considered frailty, 1–2 for pre-frailty and 0 for robustness.

### Machine learning model fitting

“Frail”, “Pre-frail,” and “Robust” were set as the output variables (Y), and the variables after univariate analysis were further filtered and embedded screened, and the final variables were used as input variables (X_n_) to fit the decision tree model. The evaluation of the three-classification model was carried out by the macroscopic method, that is, after the Y mute variable, the performance evaluation of each level and the comprehensive level were carried out, respectively.

#### Statistical analyses and quality control

The formulation of the dietary intake questionnaire was based on the General Plan for the Survey of Nutrition and Health Status of Chinese Residents [12] and was determined after evaluation and revision by relevant experts. Nutrient intake was calculated based on the 6th edition of the China Food Composition Standard Edition [13].

R software (R Project for Statistical Computing, version 4.1.3, http://www.r-project.org/) was used for the statistical analysis, decision tree model construction, and performance evaluation, and the performance of the model was evaluated by the area under the receiver operating characteristic curve (ROC), sensitivity, and specificity. The two-sided test level (α) was 0.05.

## Results

### Demographic characteristics

**Fig. 1:**
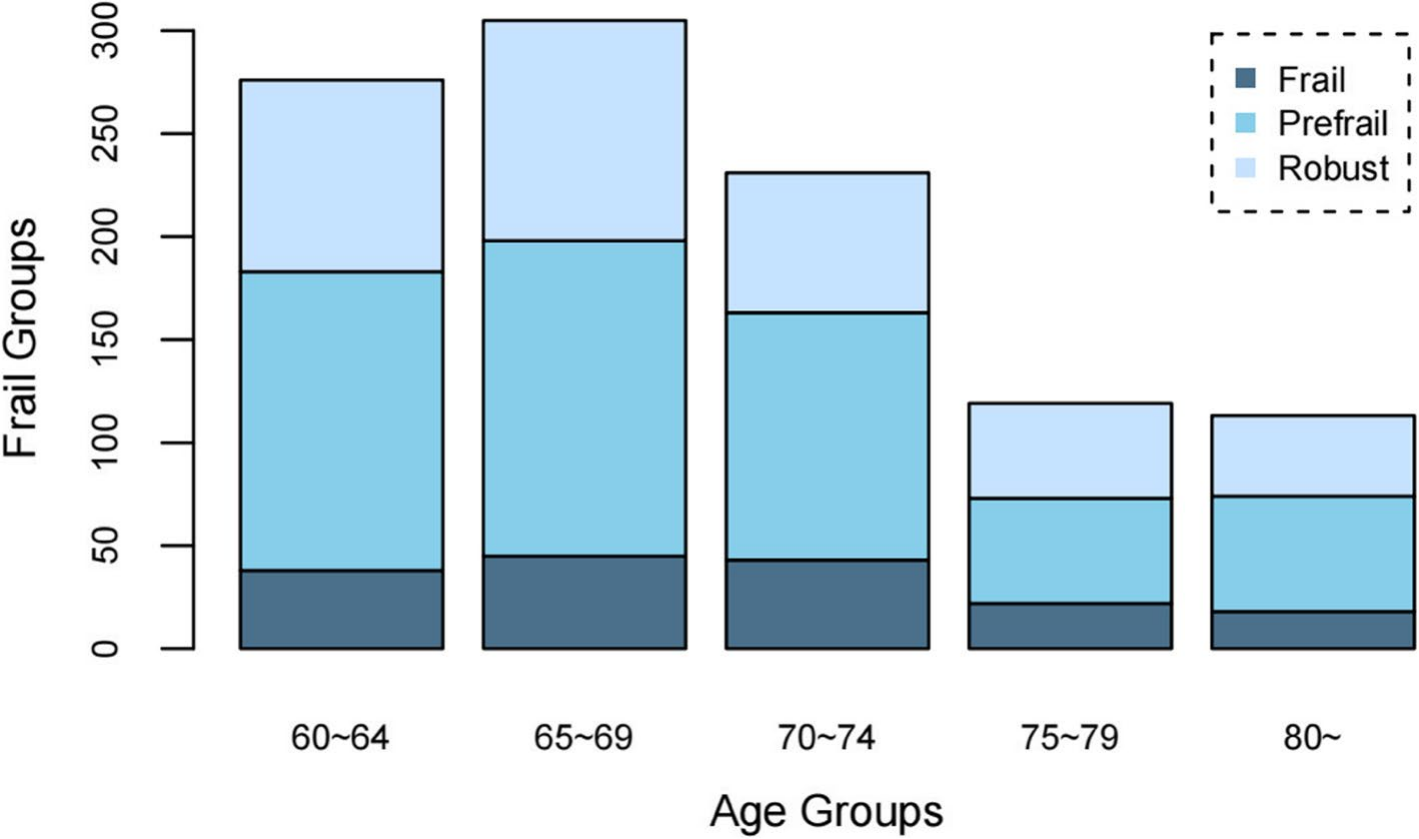
Age distribution in different frail groups

A total of 1,126 participants were surveyed, and 1,044 valid questionnaires were collected, with an effective response rate of 92.72%. The average age of the participants was 69.85 ± 6.83 years, and the oldest was 97 years old. The education level of participants was mostly at the primary school level (65.52%), occupational distribution was the highest proportion of workers and farmers (76.25%), 76.44% of participants were married, and the ratio between north and south China was 1.41:1. There were 166 (15.9%) participants with frailty, 525 (50.29%) with pre-frailty, and 353 (33.81%) who were robust (Fig. 1). The proportion of frail individuals was the largest in the 65–74-year age group (53.01%), while the proportion of pre-frail and robust individuals in the 60–69-year age group was the largest (56.76% vs. 56.66%) (Table 1).

**Table 1:** Basic demographic characteristics of 1044 research subjects

**Variable**	**Frail**		**Prefrail**		**Robust**		**Statistics**	***P***-value
	**N**	**%**	**N**	**%**	**N**	**%**		
**Age（years）**								
60~	24	14.46	65	12.38	93	26.35	46.632	＜0.001
65~	30	18.07	93	17.71	107	30.31		
70~	41	24.70	140	26.67	68	19.26		
75~	38	22.89	131	24.95	46	13.03		
80~	33	19.88	96	18.29	39	11.05		
**Education**								
Elementary school and below	137	82.53	361	68.76	185	52.41	58.734	＜0.001
Junior middle school	23	13.86	102	19.43	88	24.93		
Senior middle school	6	3.61	45	8.57	56	15.86		
College and above	0	0.00	17	3.24	24	6.80		
**Occupation**								
Worker	29	17.47	102	19.43	106	30.03	86.761	＜0.001
Farmer	108	65.06	326	62.10	125	35.41		
Cadre	4	2.41	37	7.05	63	17.85		
Self-employed	11	6.63	23	4.38	26	7.37		
Others	14	8.43	37	7.05	33	9.35		
**Marital status**								
Married	119	71.69	403	76.76	276	78.19	5.698	0.223
Unmarried	2	1.20	3	0.57	6	1.70		
Widowhood and divorce	45	27.11	119	22.67	71	20.11		
**Region**								
South China	87	52.41	277	52.76	249	70.54	30.755	＜0.001
North China	79	47.59	248	47.24	104	29.46		
**Total**	166	100.00	525	100.00	353	100.00		

Senior middle school include Secondary Special School

### Screening of factors in dietary intake and life behaviour

In this study, the factors related to frailty were investigated and analysed from the dimensions of dietary intake and individual lifestyle. We not only quantified the food intake but also converted it into nutrient intake based on the total weekly intake in the dietary dimension; 21 meaningful variables were obtained after univariate analysis (*P* < 0.05), indicating that there were significant differences in these factors among the frailty, pre frailty, and robust groups (Table 2). The lifestyle dimension covered personal lifestyle, family emotions, work, and exercise, and 12 meaningful variables were obtained after univariate screening (*P* < 0.05, Table 3). To solve the problem of collinearity between the factors of each dimension, the filter method was adopted, and nine variables (fat, dietary fibre, VA, VC, VE, CA, K, Fe, and food water content) with a correlation of more than 0.7 between the variables were preliminarily eliminated.

**Fig. 2:**
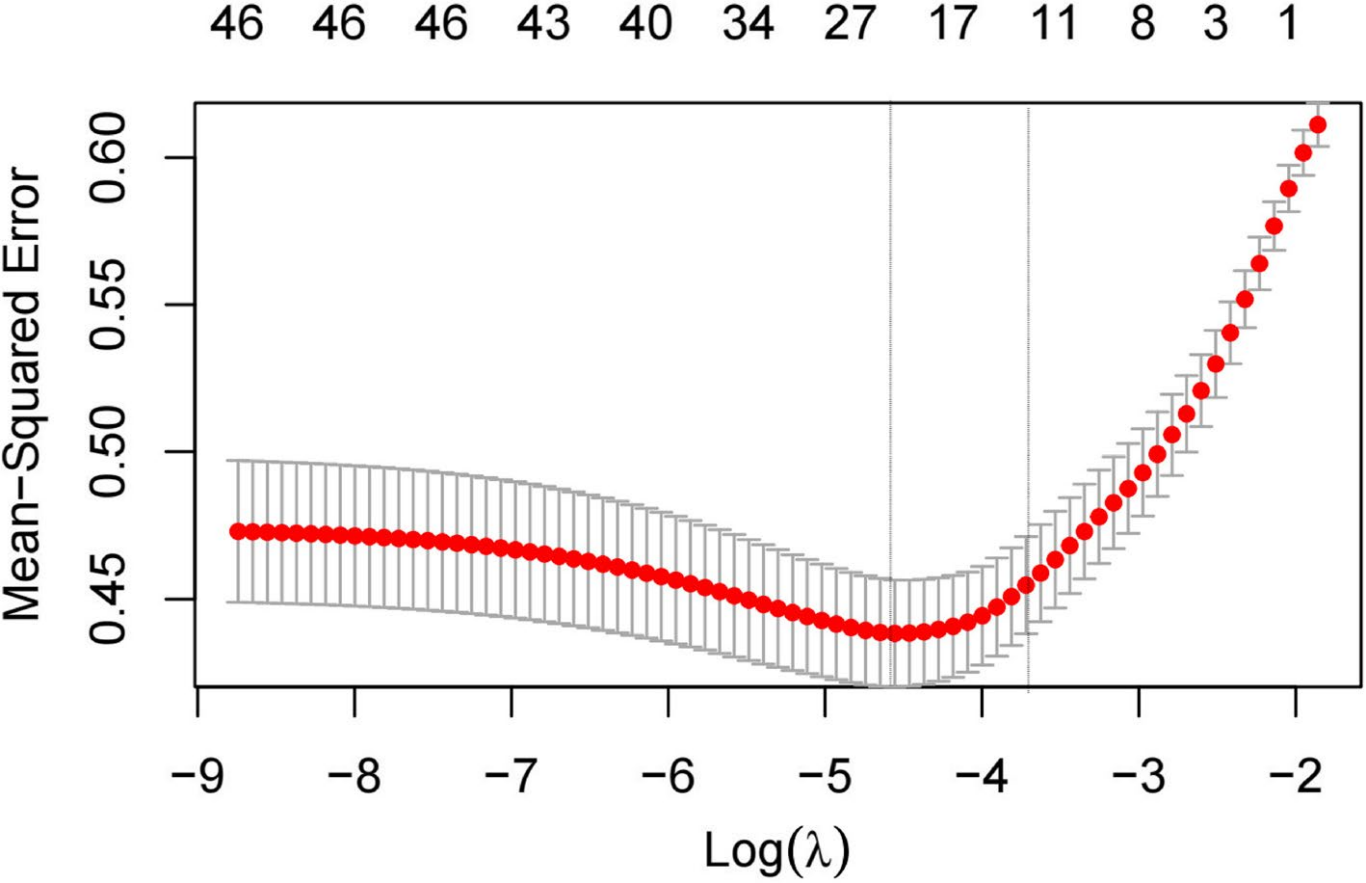
Mean-squared error for Lasso10 fold cross-verification

To explore the influence of independent variable X on dependent variable Y more accurately, the continuous variables in the remaining variables were standardised, and the categorical explanatory variables were converted into dummy variables and then screened using LASSO classification regularisation in the embedded method. The 10-fold cross-validation fitting model was used to obtain a minimum mean square error of 1.515 for the simplest model at lambda.1se (Fig. 2). Finally, eight variables closely related to frailty were identified: diet, exercise, work, and family emotions (ST1).

**Table 2:** Dietary and Weekly nutrient intake in “Frail”, “Pre-frail” and “Robust” groups

**Variable**	**Frail Groups**	**Prefrail Groups**	**Robust Groups**	**Overall comparison**	
				**Statistics**	***P***
**Staple food(**g/d**)**	419.88±90.40	446.85±91.59	378.74±93.50	6.069	0.002
**Vegetarian dishes**(**g/w**)					
Leafy vegetables	607.22±61.65	666.51±69.38	763.78±66.03	8.422	＜0.001
Root and stem vegetables	389.76±80.69	374.43±81.30	427.00±81.46	2.721	0.066
Solanaceous fruit vegetables	360.13±70.46	370.02±76.23	411.68±79.59	2.494	0.083
Melon vegetables	325.41±62.31	314.81±56.05	396.75±69.21	12.428	0.002
Legume vegetables	328.70±62.14	284.51±62.45	356.45±66.95	5.472	0.004
**Meat dishes**(**g/w**)					
Livestock	324.12±58.18	333.53±40.04	404.68±44.43	13.511	0.001
Poultry	153.27±41.68	136.08±40.88	155.57±58.55	2.131	0.119
Fish and shrimp	140.59±41.19	144.04±48.86	186.96±44.81	6.991	0.001
Eggs	220.38±58.50	223.34±55.82	255.78±56.66	4.526	0.011
**Between-meal nibbles**(**g/w**)					
Fruits	121.09±416.67	425.50±162.01	645.49±114.01	39.051	＜0.001
Milk	382.28±57.56	447.93±53.14	566.53±46.12	9.162	0.010
Nuts	90.53±43.22	111.16±43.67	121.43±43.20	2.667	0.263
**Daily** **water intake**(**ml/d**)	1199.75±244.49	1332.59±240.71	1351.78±293.12	3.485	0.031
Energy**(**Kcal**)**	13503.25±3388.5	14307.55±3933.78	13392.04±3296.52	1.723	0.423
Protein**(**g**)**	386.38±96.99	398.37±82.51	407.20±91.56	3.149	0.207
Fat**(**g**)**	239.45±97.02	257.48±95.80	289.93±95.67	4.464	0.012
Carbohydrate**(**g**)**	2522.79±500.14	2676.27±513.35	2364.07±588.46	2.62	0.270
Dietary fiber**(**g**)**	76.77±25.45	76.33±24.27	86.83±27.67	6.07	0.002
**Vitamins**					
VA**(**ug**)**	191.46±60.59	213.68±86.44	264.39±80.37	21.958	＜0.001
VB**(**mg**)**	72.11±26.40	72.29±25.02	77.77±25.09	2.859	0.058
VC**(**mg**)**	573.01±147.09	601.88±150.76	702.60±176.31	10.911	＜0.001
VE**(**mg**)**	45.23±18.45	46.22±21.77	54.26±17.91	4.279	0.014
**Macroelements**					
Ca**(**mg**)**	1533.11±378.35	1590.67±332.18	1882.12±396.75	12.389	＜0.001
P**(**mg**)**	4462.63±951.69	4673.39±922.61	4846.94±958.38	4.857	0.088
K**(**mg**)**	8273.10±1246.13	8461.19±1212.12	9151.05±1289.69	5.203	0.006
Na**(**mg**)**	1831.90±520.97	2057.07±636.63	2318.20±588.43	3.145	0.043
Mg**(**mg**)**	1421.78±479.25	1488.83±523.92	1500.84±473.54	2.697	0.260
**Microelements**					
Fe**(**mg**)**	30.61±17.28	32.00±19.03	35.51±21.20	4.839	0.008
Zn**(**mg**)**	73.63±30.44	78.69±33.86	74.34±37.52	2.064	0.356
Se**(**ug**)**	222.93±104.51	231.06±109.43	231.19±93.03	1.071	0.585
Cu**(**mg**)**	8.83±5.32	9.53±5.91	8.74±5.36	3.076	0.215
Mn**(**mg**)**	45.24±31.21	49.80±35.23	45.74±36.49	2.449	0.294
**Food** **water** **content**(**g**)	3489.11±1571.61	3588.13±1584.71	4240.09±1762.38	19.943	＜0.001
Oil**(**g**)**					
≤25	39(23.49)	131(24.95)	101(28.61)	5.618	0.467
25~50	88(53.01)	242(46.10)	152(43.06)		
＞50	27(16.27)	111(21.14)	71(20.11)		
Unclear	12(7.23)	41(7.81)	29(8.22)		
Salt**(**g**)**					
＜6	78(46.99)	231(44.00)	164(46.46)	3.951	0.683
6~10	64(38.55)	210(40.00)	145(41.08)		
＞10	18(10.84)	71(13.52)	38(10.76)		
Unclear	6(3.61)	13(2.48)	6(1.70)		
Sugar**(**g**)**					
≤25	111(66.87)	344(65.52)	249(70.54)	7.423	0.284
25~50	42(25.30)	121(23.05)	66(18.70)		
＞50	8(4.82)	45(8.57)	24(6.80)		
Unclear	5(3.01)	14(2.67)	14(3.97)		
**Spices**					
Not eat	22(13.25)	62(11.81)	32(9.07)	12.975	0.043
Eat less	50(30.12)	148(28.19)	92(26.06)		
Eat an average	60(36.14)	214(40.76)	128(36.26)		
Eat more	34(20.48)	101(19.24)	101(28.61)		

Spices refer to the intake of ginger, garlic, vinegar, peppercorns, etc.; Continuous variables are expressed in the form of (x ®±SD), statistics are F-values, and categorical variables are expressed in the form of n (%), Statistics are *χ*^2^-values

**Table 3:** Personal life behaviors and comparison in “Frail”, “Pre-frail” and “Robust” groups

**Variable**	**Frail Groups**	**Prefrail Groups**	**Robust Groups**	**Overall comparison**	
				***χ***^2^	***P***
**Dressing style**					
Pursuing fashion	3(1.81)	13(2.48)	18(5.10)	25.218	＜0.001
Follow fashion	7(4.22)	26(4.95)	35(9.92)		
Adhere to personal style	54(32.53)	113(21.52)	81(22.95)		
Not to care	102(61.45)	373(71.05)	219(62.04)		
**Duration of hobbies(h)**					
**＜**0.5	94(56.63)	258(49.14)	59(16.71)	124.929	＜0.001
0.5~1	27(16.27)	125(23.81)	132(37.39)		
1~2	29(17.47)	64(12.19)	70(19.83)		
**＞**2	16(9.64)	78(14.86)	92(26.06)		
**Emotion in life**					
Truly satisfied	59(35.54)	152(28.95)	131(37.11)	29.964	＜0.001
Satisfied	75(45.18)	274(52.19)	171(48.44)		
Not so satisfied	18(10.84)	77(14.67)	50(14.16)		
Dissatisfied	14(8.43)	22(4.19)	1(0.28)		
Satiety level					
≥90%	60(36.14)	224(42.67)	107(30.31)	14.971	0.005
70~80%	99(59.64)	271(51.62)	224(63.46)		
≤60%	7(4.22)	30(5.71)	22(6.23)		
**Sleep time point**					
Before 21:00	31(18.67)	107(20.38)	52(14.73)	26.97	0.001
21-22 o'clock	40(24.10)	198(37.71)	151(42.78)		
22-23 o'clock	54(32.53)	114(21.71)	95(26.91)		
After 23:00	27(16.27)	69(13.14)	40(11.33)		
Uncertain	14(8.43)	37(7.05)	15(4.25)		
**Night sleep duration**					
＜4	13(7.83)	52(9.90)	32(9.07)	12.311	0.055
4~6	61(36.75)	154(29.33)	90(25.50)		
6~8	73(43.98)	272(51.81)	181(51.27)		
＞8	19(11.45)	47(8.95)	50(14.16)		
**Nap duration**					
No	27(16.27)	106(20.19)	61(17.28)	16.519	0.036
＜0.5	23(13.86)	74(14.10)	49(13.88)		
0.5~	73(43.98)	185(35.24)	117(33.14)		
1~	35(21.08)	123(23.43)	112(31.73)		
**Exercise frequency**					
Occasionally	39(23.49)	239(45.52)	40(11.33)	133.569	＜0.001
Every week	17(10.24)	73(13.90)	48(13.60)		
Everyday	110(66.27)	213(40.57)	265(75.07)		
**Exercise intensity**					
Low-intensity	118(71.08)	409(77.9)	218(61.76)	35.983	＜0.001
Middle-intensity	33(19.88)	52(9.90)	81(22.95)		
High-strength	15(9.04)	64(12.19)	54(15.30)		
**Exercise duration**					
No	15(9.04)	164(31.24)	25(7.08)	199.809	＜0.001
＜0.5	43(25.90)	174(33.14)	45(12.75)		
0.5~1	72(43.37)	142(27.05)	181(51.27)		
1~2	24(14.46)	38(7.24)	82(23.23)		
**Work frequency**					
Occasionally	28(16.87)	114(21.71)	44(12.46)	18.109	0.001
Every week	18(10.84)	73(13.90)	36(10.20)		
Everyday	120(72.29)	338(64.38)	273(77.34)		
**Work intensity**					
Low-intensity	88(53.01)	235(44.76)	180(50.99)	36.639	＜0.001
Middle-intensity	44(26.51)	135(25.71)	127(35.98)		
High-strength	34(20.48)	155(29.52)	46(13.03)		
**Work self-feeling**					
Relaxed	62(37.35)	114(21.71)	143(40.51)	122.75	＜0.001
Not so relaxed	67(40.36)	247(47.05)	199(56.37)		
A little tired	23(13.86)	130(24.76)	10(2.83)		
Tired	14(8.43)	34(6.48)	1(0.28)		

Duration of hobbies refers to the time devoted to healthy hobbies everyday such as calligraphy, painting, music, fishing, dancing, raising flowers, traveling, dating, etc.; "70~80% satiety level" means that each meal eats subjectively feels hungry and full, and there is still a desire to continue eating; "≥90% satiety level" means that each meal eats subjectively feels saturated, and there is no desire to continue eating; Emotion in life refers to the comprehensive perception of the subject's emotional experiences such as husband and wife life, family member relationships, relatives and friends; Both the duration of sleep, nap, and exercise are units of hours; Low-intensity refers to walking, middle-intensity refers to brisk walking, cycling, etc., and high-intensity refers to long-distance running, swimming, fitness, etc

### Decision tree model fitting and performance evaluation

**Fig. 3:**
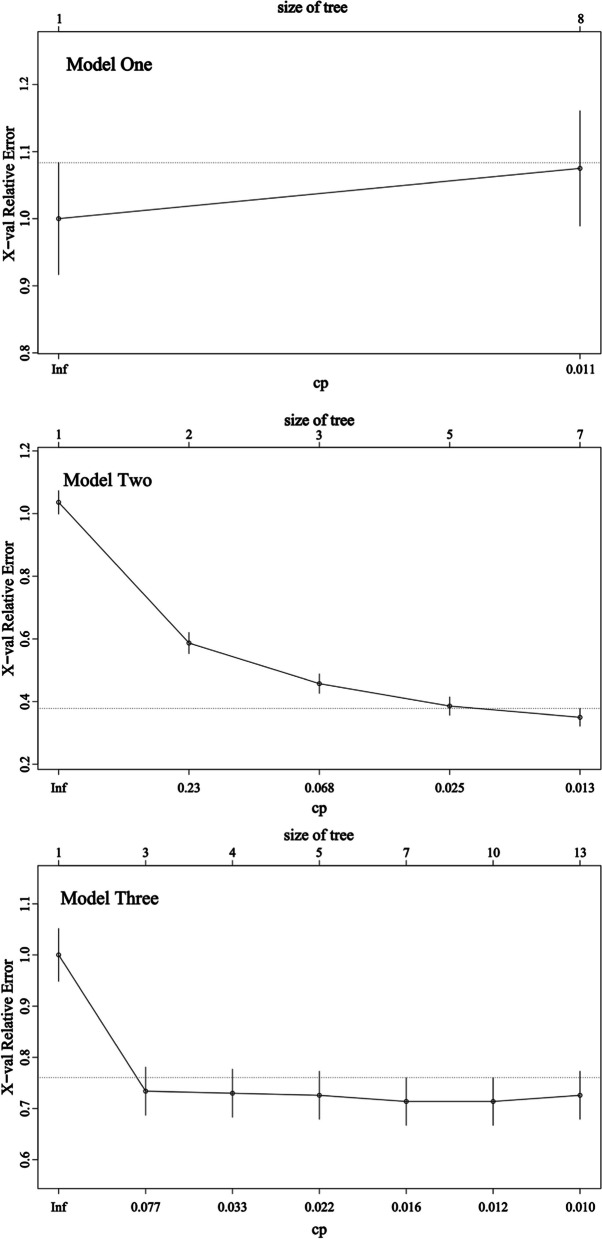
Decision tree model X-val relative error varies with the CP

The training and validation sets were randomly divided in a ratio of 7:3, and fitting and tuning were performed on the training set, followed by validation on the test set. The participants’ frailty status (Y) was converted into dummy variables (Y_1_ = frail or not, Y_2_ = pre-frail or not, and Y_3_ = robust or not), and decision tree models were fitted according to different Y_n_ values. Tree pruning was based on the Complexity Parameter (CP), and the CP value that minimised the prediction error of the model was selected (Fig. 3).

**Fig. 4:**
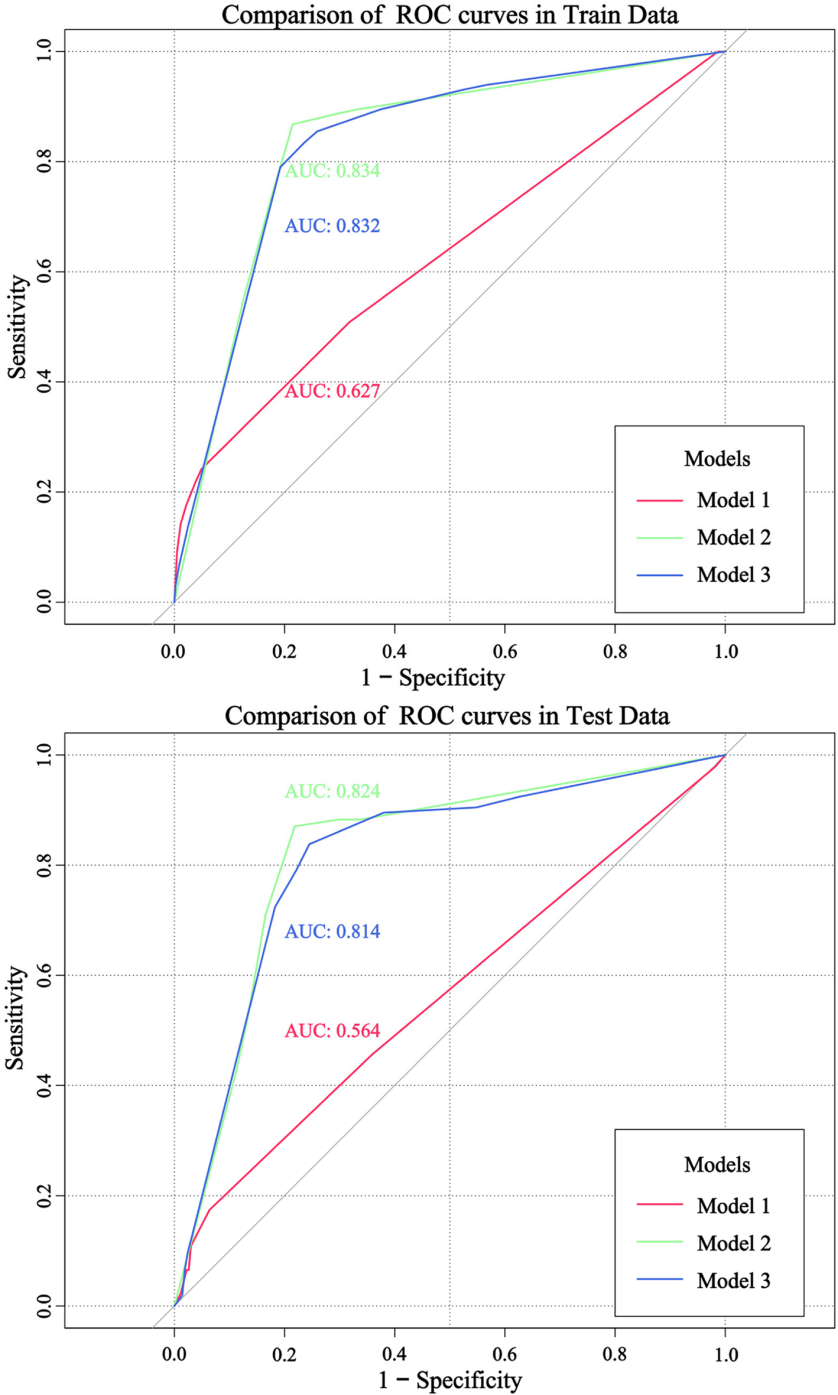
ROC curves of four decision tree model in the training set and the test set

The performance of the three models was comprehensively evaluated in the training and validation sets, and the performance of the decision tree tri-classification model was judged to be excellent from a macro perspective. Although Model 1 had high accuracy in the two sets, the recall, F1 score, and AUC were low (24.17% vs. 17.39%, 38.84% vs. 29.53%, 0.627 vs. 0.564, respectively; Table 4; Fig. 4). Based on the NPV values, the model may focus excessively on the occurrence of negative events. Importantly, the division of the dependent variable (Y) in Model 1 was extremely unbalanced, with only 15.9% assigned a value of 1(frail). The indicators of Models 2 and 3 were relatively close, but those of Model 2 were higher, but Model 2 could not distinguish the events with a value of 0 when the results were elaborated, indicating that the robust and frail people were combined, resulting in the inability to judge and distinguish the extreme effects of a certain factor on these two types of people; therefore, we used Model 3 to sort out the final results (Table 4).

**Table 4:** Performance of three classification decision tree model in training set and test set

**Data Set**	**Indicators**	**Model One**	**Model Two**	**Model Three**	
**Train Set**	Accuracy (%)	84.95	82.63	80.16	
	Recall (%)	24.17	86.78	83.47	
	Specificity (%)	95.09	78.53	76.40	
	Youden index	0.193	0.653	0.599	
	Kappa index	0.192	0.653	0.575	
	PPV (%)	49.13	79.97	64.46	
	NPV (%)	86.47	85.74	90.01	
	Precision (%)	98.85	78.53	80.75	
	F1 score (%)	38.84	82.45	82.09	
	AUC (95%*CI*)	0.627 (0.591,0.662)	0.834 (0.805,0.861)	0.832 (0.803,0.859)	
**Test Set**	Accuracy (%)	84.35	82.75	78.59	
	Recall (%)	17.39	87.04	83.81	
	Specificity (%)	93.63	78.15	75.48	
	Youden index	0.110	0.652	0.593	
	Kappa index	0.064	0.654	0.530	
	PPV (%)	31.99	81.06	63.26	
	NPV (%)	86.80	84.87	90.25	
	Precision (%)	97.75	78.15	81.73	
	F1 score (%)	29.53	82.36	82.76	
	AUC (95%CI)	0.564 (0.507,0.619)	0.824 (0.777,0.864)	0.814 (0.766,0.855)	

*PPV* Positive predictive value, *NPV* Negative predictive value

### Combined effects of dietary intake and life behaviour on frailty, pre-frailty, and robustness

**Fig. 5:**
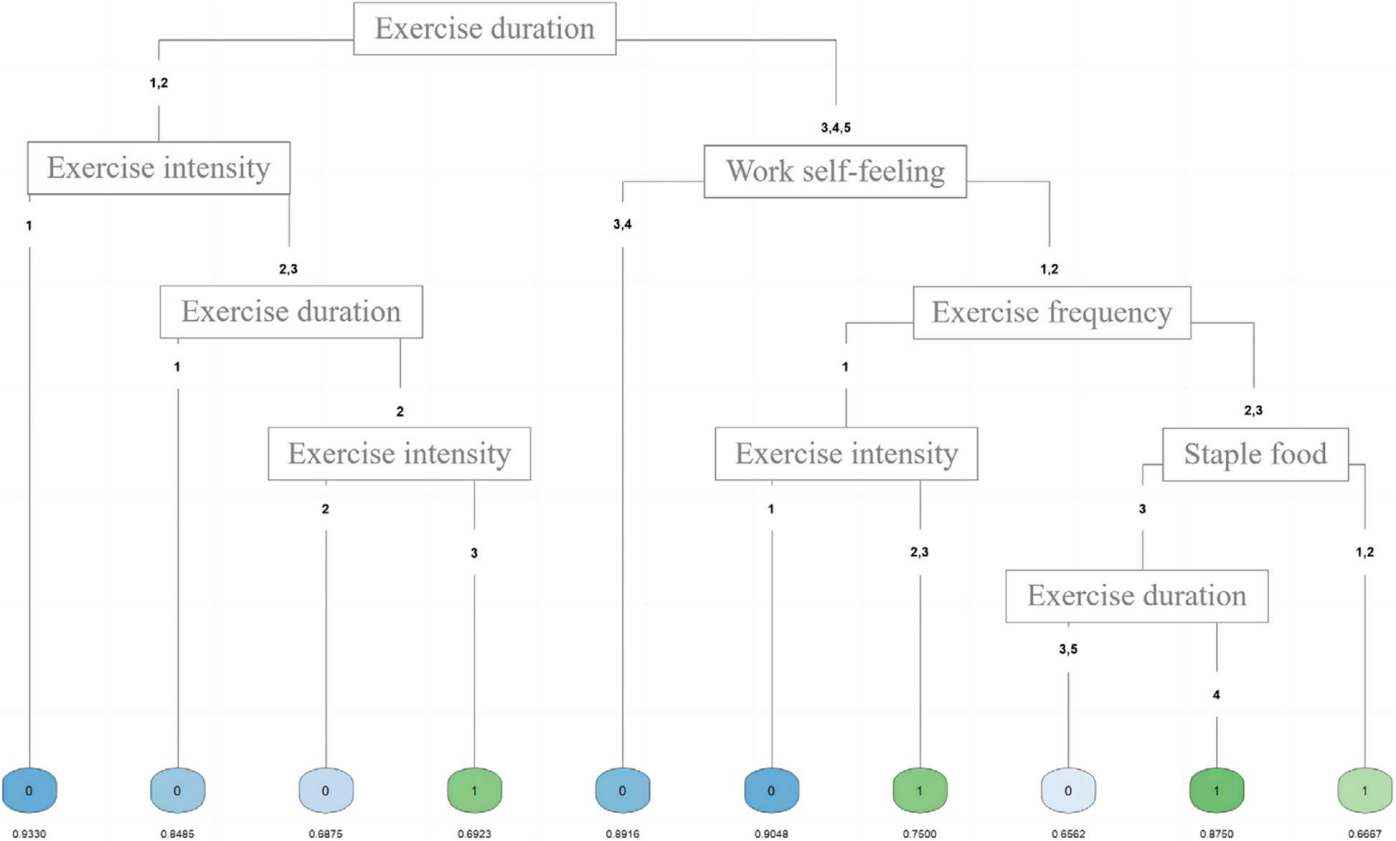
Visualization of decision tree model 3

Combined with the professional knowledge and the visualization results of decision tree model 3 (Fig. 5), we comprehensively sorted out the influence judgment of 6 variables and 10 types on robust and non-robust (Table 5, impact range 6.67–93.33%), suggesting that the combined effect of these factors is not conducive to maintaining personal robustness: not exercising or spending less than 0.5 h per day on exercise, occasional exercise with low exercise intensity, self-feeling a little tried or more at work, and excessive consumption of staple foods (> 450 g per day).

**Table 5:** Comprehensive impact of dietary intake and lifestyle behavior on “Robust” and “Unrobust” Groups

Type	Exercise duration	Exercise intensity	Work self-feeling	Exercise frequency	Staple food	Contribution rate	
						Not Robust	Robust
1	<0.5 h	Low-intensity				93.33	6.67
2	No	Middle or high-strength				84.85	15.15
3	<0.5 h	Middle-intensity				68.75	31.25
4	<0.5 h	High-strength				30.77	69.23
5	≥ 0.5 h		A little tired or tired			89.16	10.84
6	≥ 0.5 h	Low-intensity	Relaxed or not so relaxed	Occasionally		90.48	9.52
7	≥ 0.5 h	Middle or high-strength	Relaxed or not so relaxed	Occasionally		25.00	75.00
8	≥ 0.5 h		Relaxed or not so relaxed	Every week or day	<450 g	33.33	66.67
9	0.5 ~ 1/>2 h		Relaxed or not so relaxed	Every week or day	≥ 451 g	34.38	65.62
10	1 ~ 2 h		Relaxed or not so relaxed	Every week or day	≥ 451 g	87.50	12.50

## Discussion

Frailty research is a challenging topic in the life sciences. In recent years, many scholars have conducted a large number of prospective cohort studies for the treatment of frailty, and our scientific hypothesis is that by regulating the diet and lifestyle characteristics of the population before the onset of frailty, the threshold of frailty treatment can be moved forward, and the focus of medical work can be shifted to prevention and health care. After the field investigation in China, our hypothesis was confirmed: older Chinese women who do not exercise or spend less than 0.5 h a day at the same time, occasionally exercise and exercise at a low intensity, feel more tired at work, and eat too many staple foods (> 450 g per day) are not conducive to staying robust.

Prevalence of frailty in Chinese older female population.

Our study found that frailty prevalence in older Chinese women was 15.9%, which is higher than that in 2019 in China’s community of the older population [12.8%, (10.5%, 15.2%)] [5]. The increase in the value and COVID-19 has a certain relationship; most COVID − 19 poor prognosis and death occurs in older people. Clinical samples test results showed that C-reactive protein, interleukin 6, lactate dehydrogenase, calcitonin, transferrin of older samples, elevated cortisol levels were different degree of meaningful, and vitamin D levels were significantly lower (generally accepted biomarkers of frail) [14]. During the pandemic, social isolation has become a part of the daily lives of the older population in China. Family conflict, depression, anxiety, and other emotions caused by isolation reduce collective disease tolerance and further exacerbate frailty [15].

After age stratification with a span of 5 years, the overall prevalence of frailty and pre-frailty increased first and then decreased with age, showing an inverted “U” shape change. The peak of the inverted “U” appeared in the 70–74 age group, indicating that the physical injury and disease manifestation of the study participants increased in the young-older age group (60–74-year-old). Awareness and behaviour of self-care in women over 75 years of age began to increase, while the prevalence of frailty decreased.

Fitting and performance evaluation of a three-classification decision tree model.

To draw intuitive and in-depth conclusions, we focused on the detailed quantification of dietary intake and lifestyle behavioural factors that influence frailty. The constructed three-classification decision tree model focused on the systematic screening of influencing variables; that is, on the basis of single factors, filters, and embedded variable screening methods were adopted, and the variable assignment was analysed in combination with professional knowledge. The decision tree model was adopted as the auxiliary model for this analysis because its result is an intuitive and easy-to-understand tree. It can not only screen the main factors affecting frailty again, but also clearly show the interaction between factors and the contribution degree of each type of effect, avoiding the classification problem that complex parameter estimation cannot be expressed by functions [16].

In the training and test sets, Model 3 exhibited excellent performance, and the values of each index were relatively average. The accuracy, sensitivity, and specificity ranges of Model 3 were between 75% and 84%, and the AUC was greater than 0.800. The intake of fruit, milk, eggs, sedentary behaviour, and sleep time were not reflected in the final results, but this does not mean that the older adults did not need to pay attention; in fact, the final results were refined in layers of screening, the importance of variables was relatively high, and more attention needed to be paid.

Effects of dietary intake and lifestyle behaviour on population.

The dietary and lifestyle questionnaire used in this study, combined with authoritative research and surveys, retrospectively summarises the work experience of our group, which is in line with the actual situation of women. Before the survey, each investigator received uniform training and learned a clear definition of each indicator to ensure the authenticity and reliability of the results as much as possible.

Eating too many staple foods (> 450 g per day) is detrimental to robustness. Women in southern China mainly consume rice as their staple food, while women in northern China mainly consume pasta; however, both rice and pasta are the main sources of carbohydrate intake, and excessive intake of carbohydrates can lead to obesity and overweight [17]. Sarcopenic obesity is a clinically functional disorder in which obesity and sarcopenia coexist. It is characterised by a decrease in lean body mass accompanied by excessive accumulation of adipose tissue, particularly visceral fat, and is highly prevalent in older women [18]. Previous studies on frailty have focused on supplementing protein diets in diagnosed populations to regulate metabolism and improve muscle mass [19]; however, our study suggests, for the first time, that carbohydrate restriction appears to be more important than protein supplementation in healthy people (who have not progressed to frailty). Restricting carbohydrate intake has performed well in women for weight loss, insulin resistance, and blood glucose and lipids [20]; however, proper carbohydrate intake (not low-carbohydrate and high-carbohydrate diets) can increase muscle glycogen stores, muscle mass, and bone mass [21] and prevent osteoporosis. Notably, a combination of exercise and reduced carbohydrates in the diet appears to reduce the loss of muscle mass caused by ketosis [22].

Therefore, we conducted a comprehensive analysis of life behaviours, in which exercise time, intensity, and frequency were identified in the model. Further quantitative results showed that not exercising or exercising for less than 0.5 h a day, while occasionally exercising and exercising at a low intensity, does not contribute to maintaining physical robustness. Randomised clinical trials have demonstrated that exercise can significantly improve the quality of life in older adults, reduce age-related oxidative damage and chronic inflammation, and improve mitochondrial function, actin profile, the IGF-1 signalling pathway, and insulin sensitivity [23, 24]. A combination of exercise and dietary nutrition is recommended for the prevention and treatment of frailty [25].

We propose that, after maintaining good diet and exercise habits, work fatigue in older women should be reduced. Self-perception of work is a comprehensive measure of women’s psychological and social health; we define “work” as social work and family work in this study. In society, Chinese women work until the age of 55 before they can retire, and the working hours of the freelance self-employed depend on their economic pressure. In the family, women are expected to take care of their older parents, children, and grandchildren, as well as provide emotional support and household chores. Owing to the intensification of aging and the extension of women’s life expectancy, women have been in this stage for a longer period of time, increasing their energy, emotional, and economic investment. If they feel tired, it means that an increase in age and changes in social roles have increased their psychological pressure, which is also the root cause of mental illness. Frailty is a biopsychosocial syndrome [26], and epidemiological evidence suggests that more than one-third of older patients with depression [27] are frail; therefore, we do not recommend that older women remain stressed at work, as this can easily lead to psychologically related disorders and thus frailty.

Although our study is based on a cross-sectional survey, it has the following three advantages: First, our research perspective is based on the representative provinces and cities of China, and the geographical and age distribution of the research objects is uniform, which can reflect the objective reality of frailty in older Chinese women; second, based on the two dimensions of dietary intake and life behaviour, we comprehensively evaluated the comprehensive effect of the factors in two dimensions on the population simultaneously. Finally, we explain and predict the comprehensive effect of frailty in two dimensions, and the results prove the simplicity and feasibility of the medical focus from frailty treatment to frailty prevention.

This study has some limitations. The intensity of cross-sectional investigations in epidemiological studies is still low in causal judgments, and we cannot tell whether a negative combination of factors causes frailty or frailty results in a negative combination of these factors. Follow-up studies should obtain information on a larger sample. Despite these limitations, our work innovatively suggests the feasibility of shifting the focus of frailty efforts and providing positive information for day-to-day prevention of frailty.

### Supplementary Information

**Supplementary Material 1.**

## Data Availability

The datasets generated and/or analysed during the current study are not publicly available due protecting the privacy of participants but are available from the corresponding author on reasonable request.
