# Supplementary material for: Diet and lifestyle behaviours simultaneously act on frailty: it is time to move the threshold of frailty prevention and control forward
Source: BMC Public Health. 2024 Apr 20;24:1097. doi: 10.1186/s12889-024-18639-y (PMC11032589; doi:10.1186/s12889-024-18639-y)
Supplement: Supplementary file 1 — Supplementary Material 1. [file 12889_2024_18639_MOESM1_ESM.docx]

**Supplementary Table 1 Decision tree model variable assignment**

| **Variable** | **Assignment** | | | | |
| --- | --- | --- | --- | --- | --- |
| Frail Groups(Y) | Model One 1=Frail | Model Two 1=Pre-frail | | Model Three 1=Robust | |
| Staple food (X_1_) | 1=＜150 | 2=150~450 | 3=≥450 |  |  |
| Spices(X_2_) | 1=Not eat | 2=Eat less | 3=Eat an average | 4=Eat more |  |
| Exercise frequency(X_3_) | 1=Occasionally | 2= Every week | 3= Everyday |  |  |
| Exercise intensity(X_4_) | 1=Low-intensity | 2=Middle-intensity | 3=High-strength |  |  |
| Exercise duration(X_5_) | 1=No | 2=＜0.5 | 3=0.5~1 | 4=1~2 |  |
| Work frequency(X_6_) | 1=Occasionally | 2= Every week | 3= Everyday |  |  |
| Emotion in life(X_7_) | 1=Truly satisfied | 2= Satisfied | 3=Not so satisfied | 4=Dissatisfied |  |
| Work self-feeling(X_8_) | 1=Relaxed | 2=Not so relaxed | 3=A little tired | 4=Tired |  |
| The continuous variables X_1_ were transferred to categorical variables. | | | | | |
